# Review of Immunohistochemistry Biomarkers in Pancreatic Cancer Diagnosis

**DOI:** 10.3389/fonc.2021.799025

**Published:** 2021-12-20

**Authors:** Tuan Trong Luu

**Affiliations:** Management & Marketing Department, Swinburne University of Technology, Melbourne, VIC, Australia

**Keywords:** biomarker, immunohistochemistry, pancreatic cancer, sensitivity, specificity

## Abstract

Pancreatic cancer is one of the cancer types with poor prognosis and high rate of mortality. Diagnostic modalities for early detection of pancreatic cancer have been among the academic concerns. On account of the potential role of immunohistochemistry (IHC) biomarkers in overcoming certain limitations of imaging diagnostic tools in discriminating pancreatic cancer tissues from benign ones, a growing scholarly attention has been given to the diagnostic efficacy of IHC biomarkers for pancreatic cancer. This review will analyze and synthesize published articles to provide an insight into potential IHC biomarkers for pancreatic cancer diagnosis.

## Introduction

In the developed countries, pancreatic cancer is presently the fourth leading cancer cause of mortality (1). Age-standardized incident rate of pancreatic cancer is 7.2 per 100,000 in developed countries versus 2.8 in less developed regions (2). Pancreatic cancer incident rate has gradually accelerated and it is anticipated, within a decade, to increase to the second, behind lung cancer, among the leading cancer-related causes of mortality (3). Over half of the pancreatic cancer cases have been diagnosed at an advanced stage, which can partially explain its five-year survival rate being under 9% (4).

The most prevalent (around 85%) among all pancreatic cancer types is pancreatic ductal adenocarcinoma (PDAC) (5). Precise diagnosis of PDAC is required for optimal patient management. Nonetheless, current PDAC diagnosis modalities, such as endoscopic retrograde cholangiopancreatography (ERCP) and endoscopic ultrasound-guided fine-needle aspiration (EUS-FNA), demonstrate clinical limitations (6–8). Due to the potential resemblance of PDAC and benign diseases of the pancreas through imaging (9), certain challenges are encountered in differentiating them (6). Additionally, difficulties in gathering sufficient diagnostic samples as well as false positive and false negative diagnoses have limited the clinical utility of the EUS-FNA technique (6).

**Figure 1:**
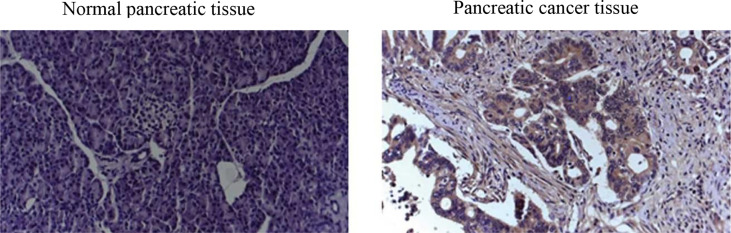
Representative IHC images in human normal and cancer tissues.

Endeavors to surmount such limitations in screening tests for early diagnosis of PDAC have concentrated on immunohistochemistry (IHC) biomarkers (3). Regardless of the proved efficacy of many IHC biomarkers for PDAC diagnosis (6) in terms of IHC images (see a representative IHC image in **Figure 1**), sensitivity, and specificity, as well as their ease of use, accessibility, and low costs (10), IHC biomarkers have demonstrated lack of consistency in diagnostic value. For instance, used as the standard pancreatic cancer biomarker, CA19-9 level elevation can be found not merely in PDAC but likewise in pancreatitis (11).

Our study hence aims to analyze and synthesize the findings from published articles in terms of the diagnostic role of IHC biomarkers for pancreatic cancer. Our review is crucial since it can suggest IHC biomarkers that are useful and efficacious for the early detection of pancreatic cancer in the clinical practices.

## Methods

### Search Strategy

A search was performed through the PubMed database to find full-text English language journal articles relevant to our research question published from 2013 up to August 2021. We chose 2013 as the lower time limit since the most comprehensive systematic review of IHC biomarkers as a diagnostic tool for pancreatic cancer was conducted by Liu and colleagues (12) in 2012. The terms used for this search strategy comprised: (cancer.mp., or neoplasms/) AND (pancreatic or pancreas) AND (biomarkers.mp., or biomarkers/, or biomarkers, tumor/) AND (immunohistochemistry/, or immunohistochemical.mp.).

### Exclusion and Inclusion Criteria

Articles that were excluded from the study consist of ones that: (1) are letters, case reports, theses, or conference proceedings; (2) merely examined IHC biomarkers in animal samples; (3) did not utilize apposite control groups; (4) demonstrated unclear clinical (diagnostic) implications; and (5) contained duplicate data or no reliable data (13, 14). No limit applied to study design, geographical area, population, or race. An article was included into the study if it contained: (1) pancreatic cancer biospecimen; (2) IHC analytical implementation; (3) clinical significance in biomarker expression; (4) apposite biomarker metrics (specificity, sensitivity); (5) univariate or multivariate analysis as statistical estimation of biomarkers for differentiation of pancreatic cancer from benign conditions of the pancreas (13).

The quality of the included studies regarding IHC biomarkers was assessed by the principal researcher and an oncologist, in light of STARD (Standards for Reporting of Diagnostic Accuracy) guideline comprising 30 items (15). An article obtained 1 point for an item if we found all its facets, 0.5 points if we found some facets, and 0 point if we did not find it. The total score of all items determines the article quality in terms of three levels: high quality: 20-30; average quality: > 10, < 20; and low quality: ≤ 10 points (14).

Journal articles included in the review met inclusion and exclusion criteria as well as the quality of average rating or above. Analyzing each included article entailed deriving the data, for each type of IHC biomarker, on the sample size, pathologically positive results, test accuracy parameters, and the link of each IHC biomarker’s expression to level of clinical evidence.

### Analytical Procedure

As aforementioned, the articles were assessed based the inclusion and exclusion criteria and the rating scores. The studies were incorporated into the review if they investigated IHC biomarkers for pancreatic cancer diagnosis and met the selection criteria as well as whether their results were inclusive or negative. The data from the articles selected were transferred into Excel spreadsheet. The characteristics of the studies embraced: (1) research title, (2) the name of the first author, (3) publication year, (4) studied IHC biomarker(s), (5) sample size (total sample size and individual sample size for pancreatic cancer, benign pancreatic diseases, and healthy/normal cases), (6) analytic techniques (IHC staining, ELISA, and/or Western blotting), and (7) statistical findings related to IHC biomarker expression (sensitivity/specificity). The articles were further analyzed in light of the findings regarding the efficacy of IHC biomarkers in discriminating pancreatic cancer from benign pancreatic diseases and normal/healthy cases.

## Results

### Included Studies

**Figure 2:**
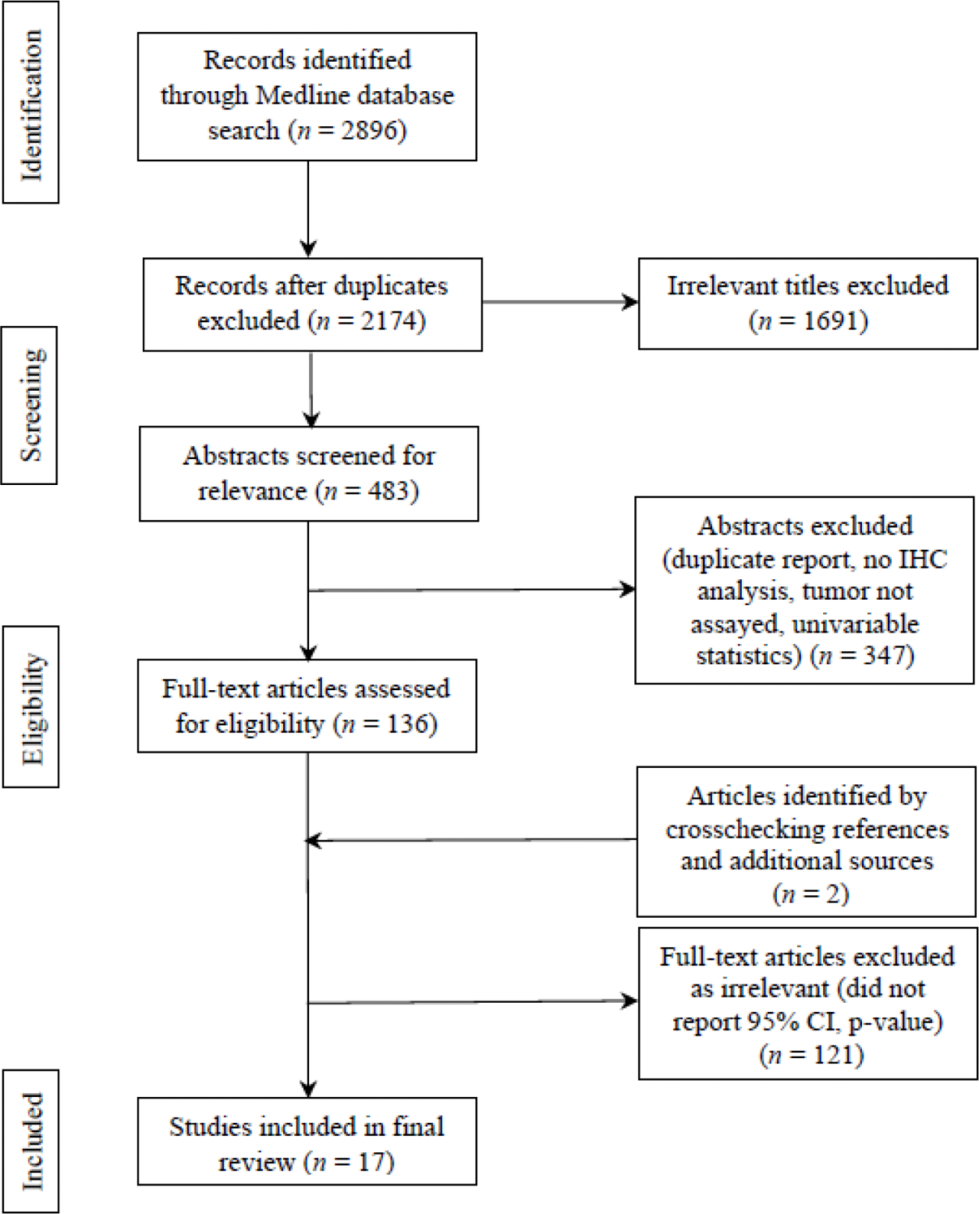
PRISMA flow chart illustrating stages of selection of articles for the systematic review.

Systematic Reviews and Meta-analyses (PRISMA) flow chart was utilized for identifying, screening, and assessing articles in light of the inclusion and exclusion criteria. PRISMA flow chart is portrayed in **Figure 2**. 2896 studies were identified at initial literature search for the period of 2013-present. Eliminating duplicates led to 2174 studies. Screening titles and abstracts yielded 483 studies that underwent the assessment of full texts for eligibility. After further excluding studies that reflected low quality based on the criteria from STARD, as well as incorporating two studies through crosschecking references, 17 articles were found to exhibit significant discriminatory values of 22 IHC biomarker(s).

### Summary of the Key Characteristics of the Included Studies

**Table 1** recapitulates the characteristics of the included studies comprising IHC biomarker(s) investigated, samples (pancreatic cancer versus healthy/normal case, pancreatic cancer versus benign pancreatic diseases especially chronic pancreatitis, or pancreatic cancer versus both), assay method(s), staining pattern(s), sensitivity, specificity, authors, and publication year.

**Table 1:** Characteristics of the included studies on IHC biomarkers for pancreatic cancer diagnosis.

IHC biomarker	Sample size	Assay(s) used	Staining pattern(s)	IHC score/threshold for positivity	Sensitivity (%)	Specificity (%)	Other IHC expression indicators	Authors and publication year
Pentraxin 3 (pentraxin family member with inflammatory properties)	140 PDAC, 86 benign pancreatic conditions, 19 gallstone disease, and 22 normal healthy	Immunohistochemistry, ELISA, and Western blotting	Cytoplasmic	Not mentioned	86	86	PPV = 97%	Goulart et al., 2021 (16)
Galectin-9 (β-galactoside-binding lectin family member with immunomodulatory attributes)	70 PDAC, 36 benign pancreatic disease, and 28 healthy	Immunofluorescence and immunohistochemistry, and ELISA		Not mentioned			c-statistic of 0.776	Seifert et al., 2020 (17)
Enolase 1 (ENO1) (pyruvate synthesis-related metabolic enzyme)	73 pancreatic cancer patients without jaundice and 50 healthy	Immunohistochemistry	Cytoplasmic and nuclear	IHC score = 12.34 ± 2.79 (PDAC) vs. 7.26 ± 3.31 (normal)	75.8	88.2		Yin et al., 2018 (18)
REG4 (regenerating islet-derived family member)	154 PDAC, 34 CP, and 74 healthy	Immunohistochemistry and ELISA		28.1% of the tumor cases: positive (positive when staining was visible)	82	79		Saukkonen et al., 2018 (19)
Periostin (POSTN) (matricellular protein)	213 PDAC, 49 CP, 26 other benign pancreatic diseases, and 74 healthy	Immunohistochemical staining and ELISA		Combining intensity and percentage of positivity (1 = weak, 2 = moderate, 3 = strong)	85.7 (PDAC CA19.9‐negative)	71.6		Dong et al., 2018 (20)
CA242 (sialic acid-containing carbohydrate antigen bound to core lipids/proteins found in serum or on the cell surface)	Same as above	Electrochemiluminescence immunoassays			69.6 (PDAC CA19.9‐negative)	68.9		Dong et al., 2018 (20)
Galectin-1 (β-galactoside-binding lectin family member with immunomodulatory attributes)	Cohort 1: 31 PDAC, 23 CP, 7 normal; Cohort 2: 28 PDAC, 27 CP, 14 normal Cohort 3: 31 PDAC, 2 CP, 7 normal	Immunohistochemistry and ELISA		Multiplying H-scores and stroma percentage	82.8	100		Martinez-Bosch et al., 2018 (21)
Maspin (B serine protease inhibitor family member)	33 PDAC and 34 control cases (3 mucinous cystic neoplasm, 8 CP, and 23 nontumoral pancreatic tissues)	Immunohistochemistry		Mainly 2+ and 3+ (Staining intensity = negative (< 5% of tumor cells stained), 1+ (5%–25%), 2+ (26%–50%), 3+ (51%–75%), and 4+ (> 75%))	87.5	100		Aksoy-Altinboga et al., 2018 (22)
on Hippel-Lindau gene product (pVHL) (hypoxia‐inducible factor family member)	Same as above	Immunohistochemistry			100	81.8		Aksoy-Altinboga et al., 2018 (22)
CA125 (sialic acid-containing carbohydrate antigen bound to core lipids/proteins found in serum or on the cell surface)	97 PDAC and 115 benign pancreatic tissue cases	Immunohistochemistry	Basolateral membrane and cytoplasmic staining	IHC ≥ 4 (43.3%), IHC < 4 (24.7%), IHC = 0 (32.0%) (IHC score = intensity × percent)	68	96.5		Jiang et al., 2017 (23)
MUC5AC (membrane-bound mucin glycoprotein)	the University of Pittsburgh Medical Center (n =321) and the Mayo Clinic (n =94)	Immunohistochemistry and ELISA		Average H-score = 1.3 ± 0.15 [H-score = percentage of cells positive × staining intensity (0–3)]	83	80	PPV = 84%	Kaur et al., 2017 (24)
Thrombospondin-2 (THBS2) (member of the matricellular Ca2+-binding glycoproteins family)	197 PDAC, 140 healthy, 115 patients with intraductal papillary mucinous neoplasm without PDAC	Immunohistochemistry, ELISA, and Western blot analysis	Membranous and nuclear	Cutoff of 2.47 for the original and cross-validation THBS2 assays	87	98		Kim et al., 2017 (25)
LTBP2 (Latent transforming growth factor b binding protein 2)	141 PDAC, 20 with benign diseases of the pancreas, and 20 normal	Immunohistochemistry and ELISA		Staining density: 0 = no staining, < 1% of cells; 1 = weak, 1-30%; 2 = moderate, 30%-70%; 3 = strong, > 70%			Significant elevation of LTBP2 levels in the PDAC tissues versus the adjacent nontumor tissues (p < 0.05)	Wang et al., 2017 (26)
CPA4 (metallocarboxypeptidase family member)	150 PDAC, 50 healthy	Immunohistochemistry and ELISA	Epithelial cells staining	86.7% of cases showed positive staining (Staining intensity: 0 = no staining; 1 = faint, < 20% of cells; 2 = moderate, 20%-40%; 3 = strong, > 40%. Negative expression: 0 or 1; positive expression: 2 or 3)	61	90		Sun et al., 2016 (27)
Insulin-like growth factor II mRNA binding protein 3 (IMP3) (RNA-binding protein needed for the processing of ribosomal RNA)	40 PDAC, 20 chronic pancreatitis, and 10 normal	Immunohistochemistry staining	Membranous or cytoplasmic	Moderate to strong immunostaining (score 2+ and score 3+) was observed in 75% of the PDAC cases Staining intensity: 1 = weak; 2 = moderate; 3 = strong	85	90	PPV = 94.4%	Ibrahim and Abouhashem 2016 (28)
Aminopeptidase N (CD13) (transmembrane metalloproteinase for epithelial polarity orientation)	204 pancreatic cancer, 48 benign pancreatic tumors, 43 CP, and 87 healthy	Immunohistochemistry staining		The total immunoreactive score (IRS) = the staining intensity × distribution (score 0-3 = negative expression, score 4-12 = positive expression)	84.3	88.2		Pang et al., 2016 (29)
Dickkopf-1 (Dkk1) (secreted Wnt pathway inhibitor)	140 PDAC, 92 control patients without PDAC	Immunohistochemistry and ELISA		Staining intensity: 0 = no staining; 1 = weak positive (< 20% of tumor cells); 2 = moderate positive (20-50%); 3 = strong positive (> 50%)	89.3	79.4		Han et al., 2015 (30)
KOC (homology domain–containing protein)	Tissue microarrays containing tumor and normal cores in 3:2 ratio, from 99 surgically resected pancreatico-biliary adenocarcinomas patients	Immunohistochemistry	Cytoplasmic	Cut-offs for positivity: 5%, 10% or 20% positive cells; IHC score: 20; moderate-strong staining intensity	84	100		Ali et al., 2014 (31)
S100P (member of the matricellular Ca2+-binding glycoproteins family)	Same as above	Immunohistochemistry	Cytoplasmic and nuclear		83	100		Ali et al., 2014 (31)
Mesothelin (glycoprotein detected on the cell surface)	Same as above	Immunohistochemistry	Cytoplasmic and membranous		88	92		Ali et al., 2014 (31)
MUC1 (membrane-bound mucin glycoprotein)	Same as above	Immunohistochemistry	Cytoplasmic and membranous		89	63		Ali et al., 2014 (31)
PAM4 (murine monoclonal antibody)	298 PDAC, 120 CP, 79 healthy	Immunoassay		Positive cutoff value of 2.4 units/mL (calculated by ROC curve statistics) Tissue labelling: 0 = negative, <1% of the labelled tissue; 1 = weak, focal labeling of 1-25%; 2 = strong, focal labeling of 1-25%; 3 = weak, diffuse labeling > 25%; 4 = strong, diffuse labeling > 25%	76	85		Gold et al., 2013 (32)

ELISA, enzyme-linked immunosorbent assays; CP, chronic pancreatitis; PPV, positive predictive value.

As shown in **Table 1**, the number of the publications that have investigated IHC biomarkers as a diagnostic tool for pancreatic cancer has increased in the past five years. 11 out of the 17 articles (64.7%) have been published in the last five years. Across the 17 publications analyzed, there are 14 articles (82.4%) studying one IHC biomarker, two studies (11.8%) examining two IHC biomarkers, and one study (5.9%) examining four IHC biomarkers. While most articles have examined IHC biomarkers in PDAC, benign pancreatic diseases (especially chronic pancreatitis), and healthy/normal cases (70.6%), three (17.6%) out of 17 studies have assessed PDAC and healthy cases only.

In the 17 articles analyzed, total sample size that consisted of both pancreatic cancer and controls ranged from 67 to 497 with 88.2% of the studies whose sample size surpassed 100 cases. When it comes to sample size for pancreatic cancer cases only, the sample size varied between 33 and 298 with 76.5% of the studies whose sample size exceeded 90 cases of pancreatic cancer. Additionally, in terms of the efficacy to detect pancreatic cancer, 15 (88.2%) out of the 17 selected studies estimated sensitivity and specificity, whereas one study employed c-statistic (17) and the other study merely assessed the elevation of IHC expression (26). Furthermore, amongst the 17 studies analyzed, all studies utilized immunohistochemistry, 10 studies (58.8%) utilized ELISA, two studies (11.8%) utilized Western blot analysis, two studies (11.8%) used these three techniques, 10 studies (58.8%) used both immunohistochemistry and ELISA, and two studies (11.8%) used both immunohistochemistry and Western blot analysis. In terms of IHC staining, two studies (11.8%) reported cytoplasmic staining only, four studies (23.5%) demonstrated cytoplasmic and membranous staining, two studies (11.8%) reported cytoplasmic and nuclear staining, and one study (5.9%) demonstrated membranous and nuclear staining.

As presented in **Table 1**, the current review study identifies 22 IHC biomarkers as diagnostic tools for pancreatic cancer that embrace: Pentraxin-3, ENO1, REG4, POSTN, CA125, CA242, Galectin-1, Galectin-9, Maspin, pVHL, MUC1, MUC5AC, THBS2, LTBP2, CPA4, IMP3, CD13, Dkk1, KOC, S100P, Mesothelin, PAM4. **Table 1** further demonstrates that, except four IHC biomarkers (CA125, THBS2, MUC5AC, Dkk1) whose efficacy in discriminating pancreatic cancer from healthy cases have not been investigated or reported, 18 remaining IHC biomarkers analyzed (81.8%) can differentiate pancreatic cancer, especially PDAC, from healthy or normal cases. 15 out of the 22 analyzed IHC biomarkers (68.2%) indicate their efficacy to discriminate pancreatic cancer from benign pancreatic diseases especially chronic pancreatitis. Specifically, pentraxin-3 can discriminate pancreatic cancer from gallstone disease; maspin and pVHL may discriminate pancreatic cancer from mucinous cystic pancreatic neoplasms; THBS2 may differentiate between pancreatic cancer and intraductal papillary mucinous pancreatic neoplasms. Analyzed IHC biomarkers that can differentiate pancreatic cancer from chronic pancreatitis encompass REG4, POSTN, CA242, Galectin-1, maspin, pVHL, IMP3, CD13, and PAM4.

On assessing positive expression of IHC biomarkers in pancreatic cancer tissue, two (11.8%) out of the 17 included studies, comprising Goulart et al. (16) and Seifert et al. (17), did not present IHC scores or thresholds for positivity. Six studies (35.3%), consisting of Saukkonen et al. (19), Aksoy-Altinboga et al. (22), Wang et al. (26), Sun et al. (27), Ibrahim and Abouhashem (28), and Han et al. (30), used staining intensity only to present IHC biomarker positive expression. Positive expression of IHC biomarkers was assessed through IHC score in Yin et al. (18), through staining intensity × percentage of positivity in Dong et al. (20), Jiang et al. (23), and Kaur et al. (24), through H-score × stroma percentage in Martinez-Bosch et al. (21), through IHC score and cut-offs for positivity in Ali et al. (31), through positive cut-off and tissue labelling in Gold et al. (32), through cut-offs for the original and cross-validation assays in Kim et al. (25), and through total immunoreactive score (IRS) in Pang et al. (29). Predictive positive value was represented in three studies (17.6%), including Goulart et al. (16), Kaur et al. (24), and Ibrahim and Abouhashem (28).

## Discussion

### Discussion of the Main Findings

IHC biomarkers can contribute to the detection of pancreatic cancer by biologically differentiating pancreatic cancer from benign forms of pancreatic diseases as well as healthy cases (31). More than 20 IHC biomarkers have been introduced as diagnostic tools for pancreatic cancer in a systematic review by Liu et al. (12) and more than 70 IHC biomarkers as its prognosticators in a systematic review by Ansari et al. (33). Among the diagnostic IHC biomarkers, carbohydrate antigen 19-9 (CA19-9) has been thus far a gold standard IHC biomarker approved by FDA for the diagnosis of pancreatic cancer (11). Nevertheless, the findings in the current review identifies 22 IHC biomarkers for pancreatic cancer diagnosis, whose sensitivity and specificity can be comparable to this standard IHC biomarker as well as, which can add to the list of diagnostic IHC biomarkers for pancreatic cancer in Liu et al.’s (2012) (12) review. As such, except for CA19-9, Maspin, pVHL, MUC1, MUC5AC, IMP3, S100P, Mesothelin found in Liu et al.’s review, the other IHC biomarkers in the current review are potential diagnostic tools for pancreatic cancer that have been validated during the period of 2013 up to now.

Across the analyzed IHC biomarkers, sensitivity varied between 61 (CPA4) and 100 (pVHL), as well as specificity ranged from 63 (MUC1) to 100 (Galectin-1, Maspin, KOC, S100P). As such, pVHL and CPA4 are the most and the least sensitive IHC biomarkers respectively for pancreatic cancer diagnosis. The most specific IHC biomarkers consist of Galectin-1, Maspin, KOC, and S100P, whereas MUC1 is the least specific IHC biomarker for the diagnosis of pancreatic cancer in the current review. Furthermore, vis-à-vis specificity and sensitivity, the best balanced IHC biomarker is maspin with sensitivity/specificity being 87.5/100. In addition, except for galectin-9 and LTBP2 whose sensitivity and specificity were not examined, as well as except for PAM4 (sensitivity: 76), ENO1 (sensitivity: 75.8), CA242 (sensitivity: 69.6), CA125 (sensitivity: 68), and CPA4 (sensitivity: 61), 15 out of the 22 IHC biomarkers (68.2%) demonstrated the sensitivity of above 80. When it comes to specificity, barring Dkk1 (specificity: 79.4), REG4 (specificity: 79), POSTN (specificity: 71.6), CA242 (specificity: 68.9), and MUC1 (specificity: 63), 15 out of the 22 IHC biomarkers (68.2%) exhibited the over-80 specificity, in which 9 IHC biomarkers (40.9%) whose specificity was 90 upwards. Through our review, promising IHC biomarkers for pancreatic cancer diagnosis in terms of sensitivity and specificity comprise: maspin (sensitivity/specificity: 87.5/100), pVHL (sensitivity/specificity: 100/81.8), KOC (sensitivity/specificity: 84/100), S100P (sensitivity/specificity: 83/100), galectin-1 (sensitivity/specificity: 82.8/100), THBS2 (sensitivity/specificity: 87/98), mesothelin (sensitivity/specificity: 88/92), and IMP3 (sensitivity/specificity: 85/90).

As for IHC biomarkers that the current review shares with Liu et al.’s (12) review, some consistencies and disparities are identified. Consistent with Liu et al.’s (12) review, the present review analysis unveils high discriminatory value for maspin (sensitivity/specificity: 87.5/100), S100P (sensitivity/specificity: 83/100), and IMP3 (sensitivity/specificity: 85/90) in diagnosis of pancreatic cancer. While Liu et al.’s (12) review indicated that MUC5AC was expressed in 67% of the PDAC cases, the current review mirrored a rather high sensitivity and specificity (83/80) for this IHC biomarker in pancreatic cancer diagnosis. Moreover, in Liu et al.’s (12) review, MUC1 expression was positive in 95% of the PDAC cases but in 50% of the normal pancreatic ducts cases, whereas our analysis indicates a high sensitivity (89) and an acceptable specificity (63). Furthermore, our review analysis identifies pVHL as a highly sensitive and specific diagnostic IHC biomarker for pancreatic cancer (sensitivity/specificity: 100/81.8), the review by Liu et al. (12) reported negative expression of pVHL in almost all PDAC cases and its positive expression in all cases of nonneoplastic acini and ducts.

### The Validity and Credibility of Our Findings

The validity and credibility of the findings in our review might have been influenced by some factors especially from primary studies. First, patient populations demonstrated high heterogeneity in terms of ethnicity, age, and disease stage. Second, lack of uniformity in measuring the expression of IHC biomarkers in the specimens might function as a potential bias. Heterogeneity in immunostaining scoring is found among the articles incorporated into this review. The review reflects a lack of a consensus-based standard in reporting cut-offs for positivity. Staining intensity (weak, moderate, and strong) is the scoring system most commonly employed in the primary studies (18, 20, 21, 23, 26–30). IHC biomarker expression levels in pancreatic cancer have also been estimated through gauging ratios of positive cells in some primary studies (18, 20, 25, 26, 28). Third, inappropriateness in matching between cases and controls as well as small sample sizes might affect sensitivity and specificity of IHC biomarkers in some selected articles (22, 28), which might affect the quality of our review analysis.

### Strengths and Limitations

This review analysis contains some strengths and limitations. This review is amongst the few in the area of IHC biomarkers that detect early pancreatic cancer through their differentiation of pancreatic cancer from benign pancreatic diseases. By identifying and comparing 22 IHC biomarkers in terms of their sensitivity and specificity, this review can indicate to clinicians sensitive and specific IHC biomarkers for pancreatic cancer diagnosis. Our analytic review can be generalized due to the clarity and rigor in its inclusion and exclusion criteria and article quality scoring, as well as no limits applied geographical area or race.

Numerous limitations can be found in this study. First, by virtue of our search strategy that involves including merely published articles and excluding abstracts or unpublished studies, publication bias in this analysis is inevitable. Second, some IHC biomarkers in some confirmatory investigations might be missed due to our incorporation of IHC biomarker studies merely from 2013 up to present. Third, this analysis does not have strong available data on account of a small number of articles that examined specific IHC biomarkers. Fourth, results from the studies that combined IHC staining technique with other techniques might not have undergone appropriate analyses due to our focus on IHC approach to biomarkers for pancreatic cancer. Fifth, the generalizability of our analysis can be enhanced if similar findings in IHC differentiation of pancreatic cancer from benign pancreatic diseases can be reproduced from larger-scale investigations into these 22 IHC biomarkers.

## Conclusion

Several attempts to improve the current diagnostic techniques for pancreatic cancer and in turn its prognosis. Our systematic review of articles in relation to pancreatic cancer identifies 22 IHC biomarkers reported to increase in plasma of the tissues of pancreatic cancer. The studies in our review indicate a practical certainty of the precise diagnosis of pancreatic cancer by utilizing these IHC biomarkers by virtue of their sensitivity and specificity that are comparable to or higher than those of CA19–9 as a current biomarker standard. Our review may hence advance the strand of research on pancreatic cancer IHC biomarkers regardless of a need for further standardizations as well as validation for the use of IHC biomarkers alone or their panels for early diagnosis of pancreatic cancer. Further investigations should be conducted in more rigorous designs with multi-center scale, large sample sizes, appropriate control groups, annotated specimens, and standardization of immunostaining scoring and IHC threshold for positivity in pathological specimens.

## Author Contributions

The author confirms being the sole contributor of this work and has approved it for publication.

## Conflict of Interest

The author declares that the research was conducted in the absence of any commercial or financial relationships that could be construed as a potential conflict of interest.

## Publisher’s Note

All claims expressed in this article are solely those of the authors and do not necessarily represent those of their affiliated organizations, or those of the publisher, the editors and the reviewers. Any product that may be evaluated in this article, or claim that may be made by its manufacturer, is not guaranteed or endorsed by the publisher.
